# Defective mitochondrial respiration, altered dNTP pools and reduced AP endonuclease 1 activity in peripheral blood mononuclear cells of Alzheimer's disease patients

**DOI:** 10.18632/aging.100810

**Published:** 2015-10-21

**Authors:** Scott Maynard, Anne-Mette Hejl, Thuan-Son T. Dinh, Guido Keijzers, Åse M. Hansen, Claus Desler, Maria Moreno-Villanueva, Alexander Bürkle, Lene J. Rasmussen, Gunhild Waldemar, Vilhelm A. Bohr

**Affiliations:** ^1^ Department of Cellular and Molecular Medicine, Center for Healthy Aging, University of Copenhagen, 2200 Copenhagen, Denmark; ^2^ Department of Neurology, Danish Dementia Research Centre, Rigshospitalet, University of Copenhagen, 2100 Copenhagen, Denmark; ^3^ Department of Public Health, University of Copenhagen, 1014 Copenhagen, Denmark; ^4^ The National Research Centre for the Working Environment, 2100 Copenhagen, Denmark; ^5^ Molecular Toxicology Group, University of Konstanz, D-78457 Konstanz, Germany; ^6^ Laboratory of Molecular Gerontology, National Institute on Aging, National Institutes of Health, Baltimore, MD 21224-6825, USA

**Keywords:** Alzheimer's disease, APE1, bioenergetics, dNTP pools, DNA repair, mitochondria, peripheral blood mononuclear cells, reactive oxygen species

## Abstract

**AIMS:**

Accurate biomarkers for early diagnosis of Alzheimer's disease (AD) are badly needed. Recent reports suggest that dysfunctional mitochondria and DNA damage are associated with AD development. In this report, we measured various cellular parameters, related to mitochondrial bioenergetics and DNA damage, in peripheral blood mononuclear cells (PBMCs) of AD and control participants, for biomarker discovery.

**METHODS:**

PBMCs were isolated from 53 patients with AD of mild to moderate degree and 30 age-matched healthy controls. Tests were performed on the PBMCs from as many of these participants as possible. We measured glycolysis and mitochondrial respiration fluxes using the Seahorse Bioscience flux analyzer, mitochondrial ROS production using flow cytometry, dNTP levels by way of a DNA polymerization assay, DNA strand breaks using the Fluorometric detection of Alkaline DNA Unwinding (FADU) assay, and APE1 incision activity (in cell lysates) on a DNA substrate containing an AP site (to estimate DNA repair efficiency).

**RESULTS:**

In the PBMCs of AD patients, we found reduced basal mitochondrial oxygen consumption, reduced proton leak, higher dATP level, and lower AP endonuclease 1 activity, depending on adjustments for gender and/or age. CONCLUSIONS: This study reveals impaired mitochondrial respiration, altered dNTP pools and reduced DNA repair activity in PBMCs of AD patients, thus suggesting that these biochemical activities may be useful as biomarkers for AD.

## INTRODUCTION

Current diagnosis of Alzheimer's disease (AD) is based on clinical examination, neuropsychological testing and brain imaging; however, a definite diagnosis can only be made by postmortem examination. Although brain imaging and cerebrospinal fluid biomarkers are applied in patients with mild or questionable symptoms to increase the level of diagnostic certainty, no definitive diagnostic tests based on peripheral biofluids are available yet. Biomarkers that reliably predict AD would greatly assist preventative and management treatments. Several reports describe alterations in peripheral blood cell DNA repair, reactive oxygen species (ROS) production and mitochondrial activities in AD patients [1-4], suggesting that these biochemical activities could potentially serve as peripheral biomarkers for early detection of AD. In fact, mitochondrial electron transport chain dysfunction and oxidative DNA damage are associated with amyloid beta (Aβ) and tau pathologies and neuronal damage in AD [5]. Because neurons have a high rate of oxygen consumption and low levels of antioxidants, they are particularly susceptible to ROS-induced oxidative DNA damage [6]. Mitochondrial dysfunction has been demonstrated in the neurons of AD patients [7] and further shown to be linked to alterations in ROS production [8]; many of these biochemical defects in the brain appear to be reflected in peripheral blood cells [9]. Reports suggest that mitochondria are central players in maintaining genomic stability by controlling a balanced supply of deoxyribonucleoside triphosphates (dNTPs) [10, 11], the substrates for DNA polymerizing enzymes. Thus, mitochondrial dysfunction in AD cells may be reflected by altered dNTP ratios (i.e. imbalance in dNTP pools). There are no published reports on dNTP pools in AD cohorts. However, recent published data from our group points to the potential utility of dNTP levels as peripheral indicators of probable disease, by revealing that low subjective vitality is linked to both a lower dCTP and higher dTTP level [12].

Deficiencies in DNA repair of nuclear and mitochondrial DNA damage have been linked to several neurodegenerative disorders [13]. Base excision repair (BER) is the main DNA repair pathway for removing oxidative DNA lesions, such as the prolific 8-oxoguanine (8-oxoG) lesion. During BER, a glycosylase enzyme (such as 8-oxoG DNA glycosylase 1; OGG1) excises the damaged base to yield an apurinic/apyrimidinic (AP) site. This AP-site is then incised by AP-endonuclease 1 (APE1). Polymerase and ligase proteins complete the repair in processes overlapping with those used in single strand break repair [14]. We recently reported that a new mouse model of AD, generated from a cross of a common AD mouse model (3xTgAD) with a mouse heterozygous for the BER enzyme DNA polymerase β (Polβ), had aggravated features of AD relative to the 3xTgAD mouse [15]: the reduction in Polβ in these 3xTg/Polβ mice induced neuronal dysfunction, cell death, and impaired memory and synaptic plasticity. This is consistent with a previous study showing that cortical neurons isolated from OGG1-deficient mice showed enhanced oxidative DNA base lesions and cell death under ischemic conditions [16].

Defective expression or function of proteins required for BER or proteins that regulate BER have been consistently associated with neurological dysfunction and disease in humans [17]. Studies report defects in BER in AD brain [18, 19] and AD lymphocytes [20, 21], and reduced capacity to remove oxidative lesions in cultured neural stem/progenitor cells as they undergo differentiation [22, 23]. Previously, we measured BER activities in brain specimens from patients with AD and from normal controls, and found that several BER enzyme activities were deficient in AD brain regions, specifically uracil incision activity (i.e. enzyme activity of uracil DNA glycosylase, UDG), single nucleotide gap filling (i.e. DNA polymerase β activity), and 8-oxoG incision activity (i.e. enzyme activity of OGG1) [19]; these results were consistent with previous reports of lower UDG activity [24] and lower OGG1 activity [25] in AD brain. However, in our study, the AP-site incision activity (APE1 activity) was not altered in AD brains relative to control brains; this differs from previous reports of increased APE1 expression in AD brain [26, 27]. Notably, only expression levels, and not APE1 activity, were reported in the previous studies. However, a study by Huang et al. [28] demonstrated that Cdk5-mediated attenuation of APE1 incision activity resulting in the accumulation of DNA damage and enhanced neuronal death in cultured cortical neurons; this suggests that the neuronal death seen in AD may in part be caused by defective APE1 activity.

In light of the above, we investigated cellular bioenergetics respiratory fluxes (to estimate glycolysis and mitochondrial respiration), mitochondrial ROS production, dNTP levels (to look for imbalance in the dNTP pools), DNA strand breaks (estimate of DNA damage) and APE1 incision activity on a DNA substrate containing an AP site (estimate of BER), as potential peripheral biomarkers of AD. We adjusted for gender and/or age, both of which are known risk factors in AD (higher risk in women is likely mostly due to their longer lifespan [29]).

## RESULTS

### Comparison of PBMC biochemical parameters in AD and control participants

Demographic and clinical characteristics of the cohort are outlined in Table 1. There was no significant difference in age between the two groups. The higher number of AD compared to controls (53 and 30, respectively) was due to practical issues. The average MMSE score of 22.9 (+/− 4.2) is indicative of the patient selection of mild to moderate AD; a score of 23 or lower, out of a maximum of 30, suggests cognitive impairment. Mean values obtained for the various parameters in control and AD groups are presented in Table 2. We measured mitochondrial oxygen consumption rates (OCRs; respiratory parameters that estimate the efficiency of mitochondrial respiration [38]; specifically basal OCR, ATP turnover, reserve capacity, maximum capacity, and proton leak), extracellular acidification rates (ECARs; parameters that estimate of the level of glycolysis [38]; specifically basal ECAR and glycolytic reserve), levels of the four dTNPs (dTTP, dATP, dGTP, dCTP), DNA strand breaks, and APE1 DNA incision activity (indicator of DNA base excision repair capability) in the seeded PMBCs. Outcomes (R^2^ and *P* values) were either unadjusted (model 1), or adjusted for gender (model 2), age (model 3) or for both gender and age (model 4). The mean values and confidence intervals for models 2, 3 and 4 were slightly altered as expected and are shown in Supplemental Tables S1, S2 and S3, respectively; the relevant outcomes (R^2^ and *P* values) based on the average values obtained for all models are shown in Table 2. Basal OCR was significantly lower in AD, after adjustment for age (model 3; *P* = .037) and after adjustment for both gender and age (model 4; *P* = .023).

**Table 1:** Participant characteristics

Characteristic	Controls	Alzheimer
Population number, N	30	53
Age (mean/SD)	66.0 (8.7)	69.2 (9.6)
Sex (M/F in percent)	40/60	43/57
MMSE (mean/SD)	-	22.9 (4.2)

Abbreviation: MMSE, Mini Mental State Examination; SD, standard deviation. As many as possible of the cohort participants were used for the various tests performed in this study (see Table 2).

**Table 2:** Levels of various biochemical parameters in PBMCs of AD and control participants, before and after adjustments for gender, age, and for both gender and age

			Model 1				Model 2†		Model 3†		Model 4†	
Variable		N	Mean (± SEM)	95% CI	R^2^	*P*	R^2^	*P*	R^2^	*P*	R^2^	*P*
Basal OCR	CAD	2543	45.39 (16.36) 38.34 (16.72)	38.77-52.02 33.30-43.40	0.041	.096	0.1	.071	0.092	.037*	0.157	.023*
ATP turnover	CAD	2543	36.85 (13.59) 33.67 (13.61)	31.42-42.28 29.53-37.81	0.013	.357	0.06	.303	0.059	.183	0.111	.140
Reserve capacity	CAD	2543	21.80 (19.06) 29.98 (23.65)	12.98-30.62 23.25-36.70	0.032	.146	0.053	.163	0.038	.213	0.061	.242
Maximum capacity	CAD	2543	67.20 (30.21) 68.32 (29.88)	55.22-79.18 59.19-77.46	0.000	.882	0.061	.965	0.036	.820	0.102	.710
Proton leak	CAD	2543	8.56 (5.93) 5.78 (4.79)	6.47-10.65 4.19-7.37	0.063	.039*	0.086	.033*	0.07	.03*	0.095	.024*
Basal ECAR	CAD	2543	5.73 (1.84) 6.39 (2.82)	4.73-6.73 5.63-7.16	0.016	.297	0.039	.287	0.05	.508	0.071	.492
Glycolytic reserve	CAD	2543	5.08 (2.42) 5.30 (2.49)	4.10-6.07 4.55-6.05	0.002	.729	0.004	.735	0.01	.873	0.012	.880
ROS production	CAD	1623	8.76 (5.01) 6.43 (2.99)	6.77-10.76 4.77-8.10	0.082	.077	0.176	.087	0.089	.069	0.181	.080
dTTP	CAD	2728	0.92 (0.85) 1.22 (0.91)	0.58-1.26 0.89-1.56	0.03	.205	0.032	.204	0.05	.264	0.05	.272
dATP	CAD	2728	8.73 (3.64) 10.66 (3.14)	7.42-10.04 9.38-11.95	0.078	.039*	0.094	.035*	0.109	.060	0.116	.055
dGTP	CAD	2728	4.87 (1.70) 5.13 (2.52)	4.04-5.71 4.31-5.94	0.004	.663	0.004	.666	0.024	.772	0.025	.797
dCTP	CAD	2728	1.50 (0.91) 1.44 (0.82)	1.17-1.83 1.11-1.76	0.001	.791	0.044	.721	0.003	.830	0.044	.711
DNA Strand breaks	CAD	2644	37.60 (8.98) 38.12 (8.49)	34.21-40.99 35.52-40.73	0.001	.808	0.012	.749	0.001	.832	0.013	.780
APE1 activity	CAD	1818	50.93 (29.81) 30.47 (26.08)	37.52-64.35 17.05-43.88	0.124	.035*	0.389	.003*	0.15	.059	0.399	.006*

Model 1, no adjustment; Model 2, adjusted for gender; Model 3, adjusted for age; Model 4, adjusted for both gender and age.

Abbreviations: OCR, oxygen consumption rate; ECAR, extracellular acidification rate; C, normal control participants; AD, Alzheimer's disease participants; N, population number; SEM, standard error of the mean.

Units: OCRs (Basal OCR, ATP turnover, Reserve capacity, Maximum capacity, Proton leak), pmol oxygen/min; ECARs (Basal ECAR, Glycolytic reserve), mpH/min; ROS production, fluorescence; dNTPs, pmol/million cells; DNA strand breaks, percent fluorescence; APE1 activity, percent incision.

* Significant difference (*P* < .05).

† Mean/SEM and 95% CI for model 2, 3 and 4 are shown in Supplemental Tables S1, S2 and S3, respectively.

**Figure 1:**
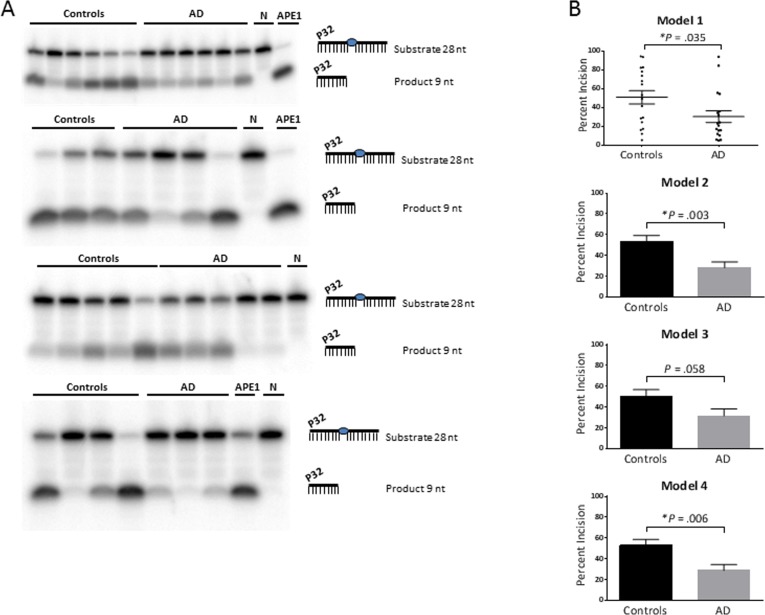
APE1 activity levels The average APE1 activity is significantly lower in the AD group relative to the control group, without statistical adjustment (model 1) and after adjustment for gender (model 2) and both gender and age (model 4); and trended lower after adjustment for age (model 3). (**A**) Gel images of radiolabeled DNA substrate and incision product, indicating the APE1 incision activities in PBMCs from AD patients and controls. The combined data from the four gels is equivalent to N of 18 for each (controls and AD). Samples were run on triplicate gels so that each value is the average of three lanes, run on separate gels; one gel from each triplicate is shown. APE1 = purified enzyme as positive control; N = negative control (no enzyme). (**B**) Comparison of APE1 incision activities in PBMCs from AD patients and controls, as generated from band intensities of 1A, and adjusted for gender and/or age. Percent incision was calculated as the amount of radioactivity in the product relative to total radioactivity per assay. Background correction was performed using no-enzyme control. *P* values were determined using the unpaired t test; the *P* values for models 2, 3, 4 above were generated in GraphPad Prism 6 using the average and SEM from Table 2. All graphs were generated in GraphPad Prism 6. Error bars represent ± SEM. As shown here and in Table 2, APE1 activity is significantly lower in AD in models 1, 2 and 4. Since model 1 has no statistical adjustments, it can be displayed as a dot plot to illustrate the raw values for each participant. *Significant difference (*P* < .05) in the average level of APE1 activity between the controls and AD.

Proton leak was significantly lower in AD, with or without the adjustments (*P* = .039, .033, .030, .024 for models 1, 2, 3, 4, respectively). The dATP level was significantly higher in AD, without adjustment (model 1; *P* = .039) and after adjustment for gender (model 2; *P* = .035). The APE1 activity was significantly lower in AD, with no adjustment (model 1; *P* = .035), adjustment for gender (model 2; *P* = .003) and adjustment for both gender and age (model 4; *P* = .006). Gel images of the APE1 activity assays are shown in Figure 1A. The graphical form of the APE1 data for each model is shown in Figure 1B; since model 1 has no statistical adjustments, we were able to display the results as a dot plot to illustrate the raw values for each participant.

### Effects of gender, age and MMSE on the parameters

**Figure 2:**
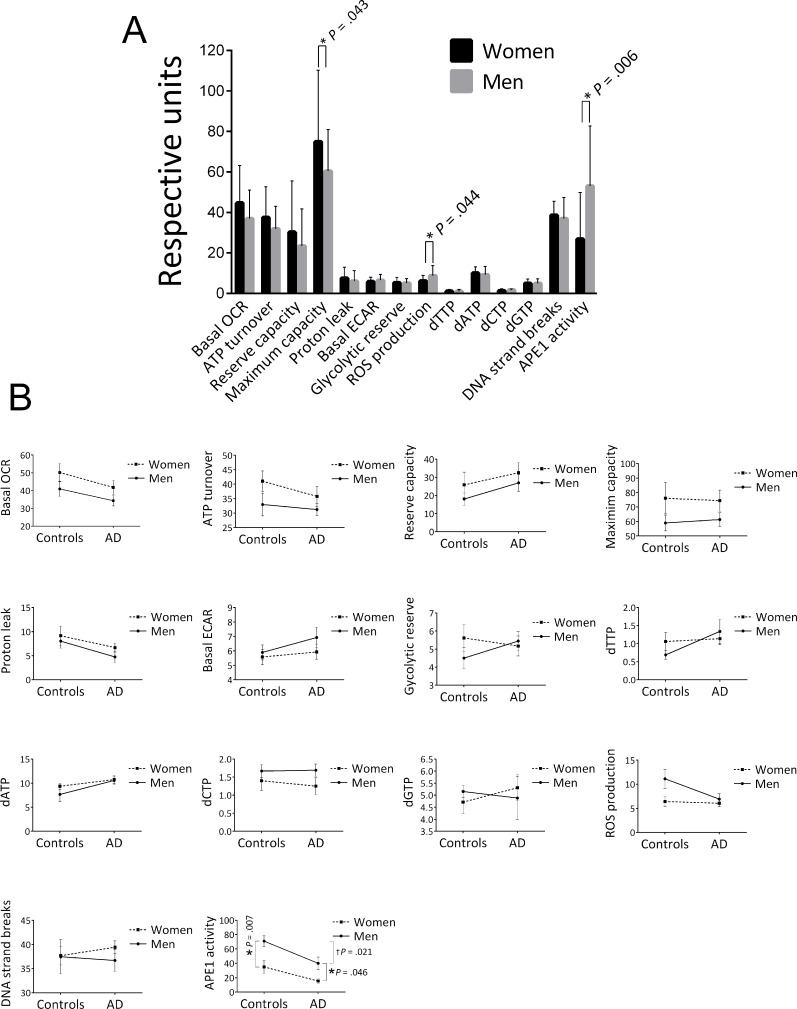
Effects of gender on the average values of the biochemical parameters (**A**) Stratified for gender. Population numbers are as follows: OCRs (Basal OCR, ATP turnover, Reserve capacity, Maximum capacity, Proton leak) and ECARs (Basal ECAR, Glycolytic reserve), men = 33, women = 35; ROS production, men = 18, women = 21; all dNTPs, men = 22, women = 33; DNA strand breaks, men = 31, women = 39; APE1 activity, men = 19, women = 17. Error bars represent ± standard deviation. * Significant difference (*P* < .05) in the average level of the parameter between men and women. (**B**) Stratified for both gender and control/AD. Population numbers in the order of control/men, control /women, AD/men, AD/women for each parameter were as follows: all OCR and ECARs, 13, 12, 20, 23; all dNTPs, 10, 17, 12, 16; ROS production, 8, 8, 10, 13; DNA strand breaks, 10, 16, 21, 23; APE1 activity, 8, 10, 11, 7. Error bars represent ± standard deviation. *Significant difference (*P* < .05) in the average level of the parameter between men and women.

We also stratified the data of the cohort into two groups, men and women, and found that women had a significantly higher mitochondrial maximum capacity, lower ROS production and lower APE1 activity (Figure 2A). In addition to the significant effect on maximum capacity, there was an apparent trend for higher levels of the other four OCR parameters in women. We also stratified for both gender and control/AD (Figure 2B); crossover of the trend lines, or large differences in the slopes of the lines, would suggest that the differences in the parameter levels between men and women that we saw in Figure 2A may be group (control, AD) specific, or that the differences in the parameter levels between control and AD that we saw in Table 2 may in fact be gender specific. This approach reduces the statistical power due to lower population numbers, and in fact we found significant differences by this approach only in the case of the APE1 incision parameter, as follows. The significantly lower APE1 activity observed in women for the entire cohort (control plus AD) (Figure 2A) was recapitulated in the control group (*P* = .007) and the AD group (*P* = .046) (see Figure 2B) and thus is not strongly group specific. We also found a significant difference in the APE1 level between controls and AD in the case of men (*P* = .021), but the difference was not significant in the case of women (*P* = .082), suggesting that the lower APE1 activity in AD as tabulated in Table 2 may have some degree of gender specificity. This is consistent with the improved *P* value obtained when we corrected for gender in Table 2 (*P* =.003 compared to unadjusted *P* value of .035). The trend lines for the other parameters, although showing no statistically significance, may be informative. Notably, in the case of each of the OCR parameters, the slopes of the men and women lines are similar, indicating that the trends for higher OCRs in women that we see in the “control plus AD” cohort of Figure 2A are not specificfor control or AD groups. In the case of glycolytic reserve, dTTP, and dGTP, the trend lines have obvious crossover, indicating group (control, AD) specificity; such cross-overs indicate that the gender-specific differences in the “control plus AD” cohort (Figure 2A) for these parameters are blunted by the use of both controls and AD in the Figure 2A analysis. In the case of ROS production there was no obvious difference between men and women in the AD group, however, there was an apparently higher level in men in the control group (Figure 2B). These ROS production trend lines, albeit not significant, suggest that the lower ROS production in women seen in Figure 2A may be mediated by the control participants.

**Figure 3:**
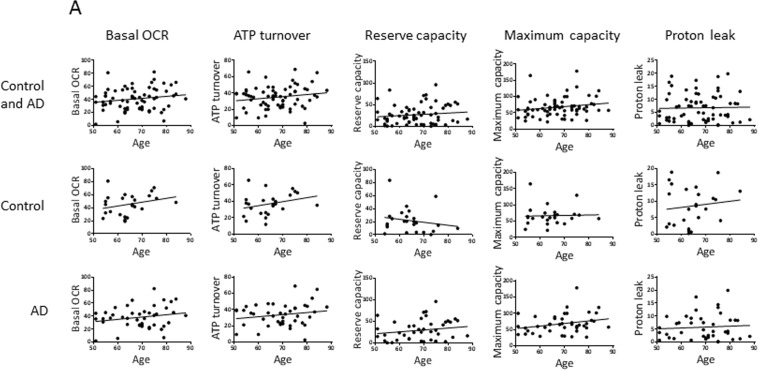

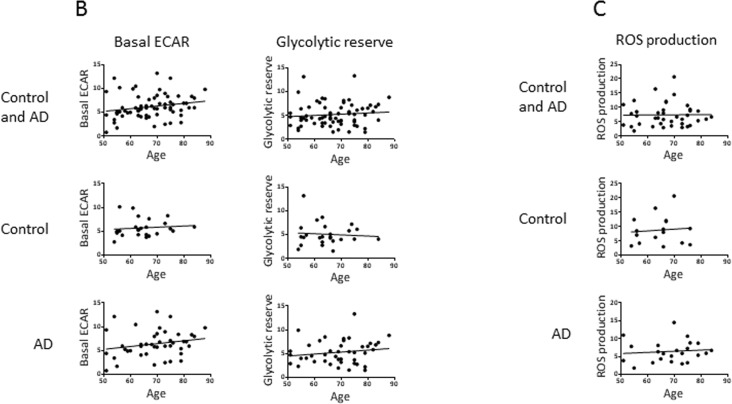

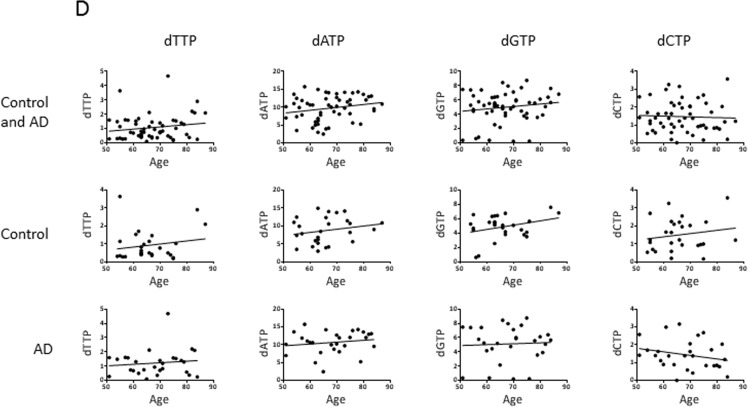

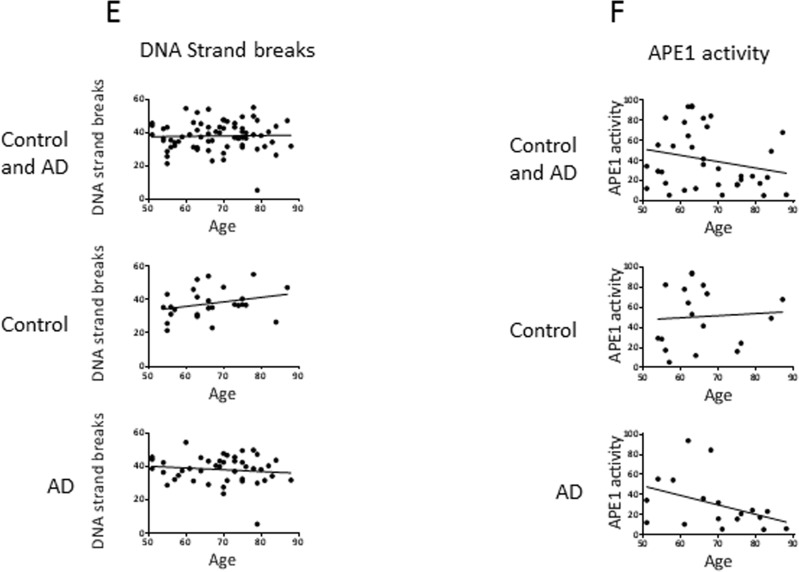
Effects of age on the values of the biochemical parameters in controls and AD participants Pearson correlation analysis of age with (**A**) OCR parameters, (**B**) ECAR parameters, (**C**) ROS production, (**D**) dNTPs, (**E**) DNA strand breaks, (**F**) APE1 activity. Units: OCRs (Basal OCR, ATP turnover, Reserve capacity, Maximum capacity, Proton leak), pmol oxygen/min; ECARs (Basal ECAR, Glycolytic reserve), mpH/min; ROS production, fluorescence; dNTPs, pmol/million cells; DNA strand breaks, percent fluorescence; APE1 activity, percent incision.

**Figure 4:**
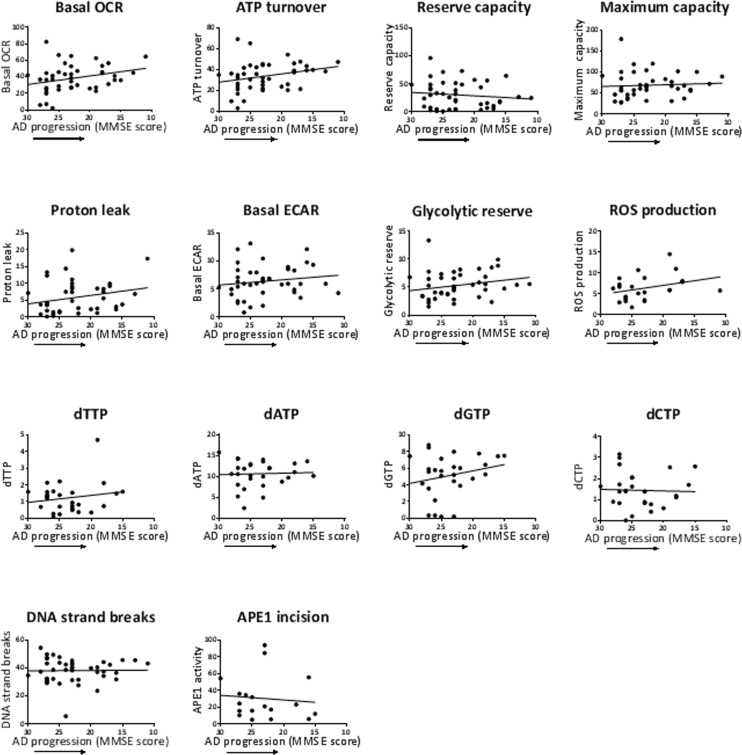
Effects of MMSE of the AD patients on the values of the biochemical parameters Pearson correlation analysis of MMSE with the biochemical parameters, as indicated. MMSE scores are listed high to low on the x-axis to correspond to AD progression (reduction in cognitive function as measured by MMSE). Units: OCRs (Basal OCR, ATP turnover, Reserve capacity, Maximum capacity, Proton leak), pmol oxygen/min; ECARs (Basal ECAR, Glycolytic reserve), mpH/min; ROS production, fluorescence; dNTPs, pmol/million cells; DNA strand breaks, percent fluorescence; APE1 activity, percent incision.

We also performed correlation analysis to investigate potential associations of the parameters with age (Figure 3) and with disease progression (MMSE score) (Figure 4). In Figure 3, the aim of the analyses within the control group was to give insight into potential biochemical links to age, independent of AD pathology; the analyses within the AD group will then indicate if age trends interact with AD pathology. There was no significant association of any of the measured parameters with age of the participants (within the entire group or within the controls or AD separately), or with MMSE score in the AD patients. However, the trends may be informative. Notably, there is a trend for higher levels of basal OCR and ATP turnover with increased age (in the control and the AD groups with a similar slope of the trend lines, indicating no interaction of AD pathology with the age trends) and disease progression (lower MMSE score). This suggests that the PBMCs may be compensating for both age and AD development by increasing the mitochondrial production of ATP. There was also a trend for higher proton leak with increased age (in the control group; the age effect on proton leak was blunted in the AD group) and with lower MMSE, suggesting that PBMCs may be compensating for age and AD development by fine-tuning respiration efficiency (see discussion). In the case of glycolysis parameters (basal ECARand glycolytic reserve) there was no obvious trend with age in the control groups, but both parameters trended to higher levels with lower MMSE, suggesting that glycolysis is compensating higher with disease progression (but not with age). ROS production was strikingly unaltered with age, but did show a trend for higher levels with lower MMSE. All four dNTPs, as well as DNA strand breaks and, to a very small degree, APE1 activity, had higher levels with advanced age in the control group; these trends were blunted within the AD group. The dGTP and dTTP parameters showed an increase with disease progression, but dATP, dCTP, DNA strand breaks and APE1 activity parameters did not; especially notable is the lack of dATP association with MMSE score since we did see a significantly higher dATP level in the AD group in Table 2. The DNA damage and repair (APE1 activity) trends indicate that DNA strand breaks increase with age (but not with disease progression) and the cells attempt to compensate with increased BER.

### Correlation analyses among the biochemical parameters

To examine links between the biochemical activities, we performed correlation analysis between all the parameters measured in this study (Supplemental Table S4). Consistent with our previous correlation analysis in PBMCs of normal (healthy) participants [12], where we compared many of the same biochemical parameters that we examine in this current report, we found that there were significant associations among the bioenergetics parameters and among the dNTP levels. These associations are to be expected [10, 39, 40] (also see Figure Supplemental S1), assuming that the parameters are measured accurately. This helps verify the specificity of our data for the biochemical activities being investigated (for example, a participant with high basal OCR, should also have a high maximum capacity OCR if the mitochondrial flux modifiers were added correctly). Focusing on the parameters that gave a significant outcome in Table 2: basal OCR was significantly associated with ATP turnover, maximum capacity, proton leak and glycolytic reserve (all with *P* < .0001); proton leak was additionally associated with ATP turnover (*P* = .007) and maximum capacity (*P* = .0068), but not with glycolytic reserve (*P* = .9499); dATP was not associated with any of the other three dNTPs, yet the other three were associated with each other (all with *P* < .005). APE1 activity was not associated with any of the other parameters.

## DISCUSSION

### APE1 activity in AD

The gender adjusted outcome for APE1 activity (significantly lower activity in the AD group) stood out as particularly compelling since it showed the strongest statistical result relative to any other parameter (R^2^ = 0.389, *P* = .003). Correction of APE1 outcome for both gender and age did not improve the strength of this outcome (R^2^ = 0.399, *P* = .006). This relatively strong outcome, does not appear be linked to the other parameters, since the level of APE1 activity was not associated with any of the parameters (Supplemental Table S4). Studies report an increased level of the APE1 protein in AD brain [26, 27]; it has been suggested that this supports the view that AD brain compensates or adapts to higher oxidative stress by increasing the level of APE1 in an attempt to better repair damaged DNA. However, here we find lower level of APE1 activity, suggesting that BER efficiency is reduced in AD PBMCs, which in turn would contribute to accumulation of oxidative lesions. Published data from our group has reported a lower activity of the BER enzymes OGG1 and DNA polymerase β, but no change in APE1 activity, in AD post mortem brain tissue, relative to controls [19]. However, our finding in this current report is consistent with several studies reporting evidence of reduced BER capacity in peripheral cells (blood cells or dermal fibroblasts) or postmortem brains of AD patients [18, 20, 25, 41-44]. Moreover, a recent study reported reduced AP-site incision activity of APE1 in addition to reduced APE1 protein level upon differentiation of neuronal cells; OGG1 and UDG activities were also reduced, however the levels of these BER enzymes were not significantly altered [23]. Inconsistencies among studies, regarding activity and levels of BER components, may be a reflection of tissue-specific differences in the individual steps in BER, for example in peripheral blood compared to the central nervous system. If our data is reproduced in follow-up studies, APE1 activity could potentially be incorporated into a DNA repair panel of risk factors for AD risk assessment or early detection, as suggested in the case of lung cancer [45]; in that study, the suggested risk factor panel consists of enzymatic DNA repair activities (in PBMCs) of APE1, OGG1, and methylpurine DNA glycosylase.

### Mitochondrial respiration in AD

The outcome for proton leak (significantly lower proton leak in AD PBMCs in all models) is intriguing since optimal proton leak is important in thermogenesis, protection against reactive oxygen species, endowment of metabolic sensitivity and maintenance of carbon fluxes [46]. Our data on proton leak suggests that AD patients are not well equipped at using proton leak to adjust respiration in response to mitochondrial stress or changes in ATP demand. The age adjusted (model 3) and the gender and age adjusted (model 4) outcomes for basal OCR suggest that AD PBMCs are deficient in basal mitochondrial respiration. This data is consistent with a recent study by Leuner et al [3], who reported a reduced basal rate of respiration in AD lymphocytes using the Oroboros Oxygraph-2 k system. In contrast, two previous reports found no changes in the enzyme activity of the respiratory chain complexes in AD lymphocytes [47, 48]. This suggests that perhaps measurement of bioenergetic fluxes is a more sensitive biomarker for AD compared to enzyme activity of respiratory chain complexes. It has also been suggested by Leuner et al. [3] that AD severity (MMSE level) may in part explain differences found between studies.

According to mitochondrial flux circuitry, a lower basal OCR could be due to a decrease in ATP turnover (reduced ATP demand), or a decrease on proton leak (with corresponding increase in membrane potential) [40]. This agrees with our data in which AD PBMCs displayed a significantly lower proton leak and a trend for lower ATP turnover (Table 2). The lower proton leak could be due to lower uncoupling protein activity and in fact this has been demonstrated in plasma of AD patients [49]. However, lower basal OCR and accompanying lower proton leak (more coupled, higher membrane potential) would be predicted to be accompanied by an increase in ROS [1] due to a more reduced electron transport chain [50]. In fact, Leutner et al [4] reported enhanced ROS production in AD lymphocytes (measured by the intracellular fluorescence dye dihydrorhodamine123). However, in our study we did not observe a higher ROS production in AD PBMCs (measured by MitoSox red), and in fact the trend was for lower ROS production in the AD PMBCs. It is possible that the mixture of white blood cells that make up PBMCs (lymphocytes, monocytes, macro-phages) dilute any trend in lymphocytes alone. Also, it has become apparent that mitochondrial ROS production can be regulated independently of oxygen consumption in many tissues and in different physiological situations, such as aerobic exercise bouts, chronic exercise training, hyperthyroidism and dietary restriction [51]. Interestingly, within the AD group there was a trend for increased ROS production with disease progression (Figure 4). We speculate that the participants within the AD group have more consistent physiological situations and thus ROS production would be more closely associated with oxygen consumption, however, more work needs to be done to assess the impact of tissue and physiological conditions on mitochondrial ROS production.

Our data indicating reduced basal OCR in AD is consistent with the underlying theme of the “mitochondrial cascade hypothesis” for sporadic AD in which it is proposed that inherited electron transport chain gene combinations determine basal mitochondrial respiration rate and persons with low rates may be at higher risk for AD [52]. The theory has come about to a large part based on the findings that amyloid β is transported to the mitochondria and binds respiratory complex IV where it is thought to then inhibit mitochondrial oxygen consumption [53]. However, the theory also proposes that the mechanism involves increased mitochondrial ROS and compensatory glycolysis stemming from mitochondrial dysfunction. Our data in Table 2 does not support this aspect of the model, since we do not see a significant increase in ROS production or basal ECAR in AD; however, both of these parameters show a trend for higher levels with disease progression (Figure 4).

### dNTPs in AD

The altered dATP level in AD, without significant alteration in the other three dNTPs, implies an imbalance in dNTP pools that may reflect mitochondrial dysfunction [39]. This finding is consistent with our correlation analysis indicating that dATP was not associated with any of the other three dNTPs, which were all associated with each other (Supplemental Table S4). There are no previous reports on dNTP imbalance in AD. However, studies suggest a model in which dTNP imbalance leads to mitochondrial DNA mutations, which are known to contribute to a range of human diseases, including neurodegenerative disorders, heart conditions, and cancer [11, 54]. Also, dATP is critical in feedback inhibition of ribonucleotide reductase (RNR), a key enzyme in de novo dNTP synthesis [55, 56]; this is important for optimal coupling of dNTP production to utilization. Since the enhanced dATP level is not accompanied by reduced levels of the other dNTPs, it appears that dATP feedback inhibition process is impaired in AD patients. However, we cannot declare in this study the reason for the enhanced levels of dATP in AD PBMCs.

### Gender effects

Within the entire “control plus AD” cohort, there was an obvious trend for higher OCRs in women relative to men (Figure 2A) and there was in fact a significantly higher level of the maximum capacity OCR parameter in women. These trends were also apparent within both controls and AD as separate groups (Figure 2B). There are limited publications comparing mitochondrial bioenergetics in men and women, but a recent study using the oxygraph respirometer found no significant association between mitochondrial respiratory parameters and gender in intact and permeabilized platelets [57]. However, there are many differences in their approach compared to ours that may explain the differences in outcome, such as the specific type of blood cells, apparatus for measuring mitochondrial bioenergetics, and participant characteristics (ours includes AD patients, and all are over 50 years of age).

There was also a significantly lower APE1 activity in the women relative to men in the entire cohort (Figure 2A), and in both the control and AD groups (Figure 2B). This is consistent with the results from a recent study by Slyskova et al. [58], in which DNA repair (both BER and nucleotide excision repair) was lower in women relative to men, in PBMCs. We also found that the average ROS production value was significantly lower in women (Figure 2A). Based on these data, we speculate that in men we see a compensation for higher ROS-induced DNA damage via enhancement of DNA repair.

### Effects of age and MMSE

The trend-lines described in the Results (parameter values in participant PBMCs versus age or MMSE; Figures 3 and 4) suggest the following biochemical events in the PBMCs: 1. Accumulation of DNA damage (DNA strand breaks) with age in healthy individuals, 2. Increased ROS production with disease progression, 3. Compensation by the mitochondrial flux parameters basal OCR, ATP turnover, and proton leak with age in healthy individuals, and with disease progression. 4. Compensation with disease progression by the glycolysis parameters, 4. Compensation by APE1 activity (DNA repair) with age in healthy individuals, 5. The trend for higher dNTPs with age in healthy individuals (all four dNTPs) or disease progression (dGTP and dTTP) may represent a response to genotoxic stress [56], and/or defective dATP feedback inhibition of ribonucleotide reductase, with age or disease progression (see above, Section 4.3). However, none of these trends reached statistical significance; note that the analysis *among* controls or *among* AD involved relative smaller population numbers, compared to the analysis *between* controls and AD of Table 2, resulting in diminished statistical power.

The aim of the MMSE correlation analysis was to see if the parameters that showed a statistically altered mean value in AD PMBCs (Table 2) (basal OCR, proton leak, dATP, APE1 activity) also showed an association (or trend) with disease progression (lower MMSE score). Basal OCR and proton leak levels in AD trended towards higher levels with disease progression; however the mean values of these mitochondrial flux parameters were significantly lower in the AD group compared to the control group (depending on gender/age adjustment) (Table 2). We speculate that basal OCR and proton leak are considerably reduced early before significant AD neuropathology, and then the cells attempt to compensate higher (to generate more ATP) as the disease progresses to more advanced stages as measured by reduced cognitive function (reduced MMSE scores). Future analyses to test and expand on this hypothesis, could entail measuring these cellular mitochondrial flux parameters in groups of high (or normal) MMSE and significantly declined MMSE score (without significant AD neuropathology), in addition to groups of control (or early AD) and severe AD (severe neuropathology). The dATP level did not show an obvious trend with disease progression; yet the mean value of dATP was significantly higher in the AD group compared to the control group (depending on gender/age adjustment) (Table 2). This suggests that dATP alteration may also be an early event, and in this case not further altered with disease severity. APE1 activity weakly trended lower with disease progression, in accord with the lower mean values in AD compared to controls (depending on gender/age adjustment) (Table 2). Notably, a recent study by Simpson et al. [59] demonstrated an increased expression of DNA damage response (DDR)-associated (double strand break repair) proteins γH2AX and DNA-PKcs associated with lower MMSE score, in the frontal neocortex of participants at the earliest stages of AD pathology, before appreciable AD pathology. In contrast, we observed a reduced activity of the DDR protein of our study, i.e. APE1, in AD PBMCs. However, our study differed in several ways from that study: assays were performed on peripheral blood cells; APE1 is associated with a different aspect of the DDR than the DDR factors of the above study, namely BER; the AD patients were of a later stage of disease (diagnosed as mild to moderate AD) where AD pathology may impede with the ability of cells to produce a vigorous DDR (such as impairing the APE1 enzyme activity).

Several studies dealing with markers of oxidative DNA and RNA damage in early stage AD neurons and association of these markers with progression of Aβ plaques and neurofibrillary tangles, suggest that oxidative damage to DNA and RNA is greatest early in the disease, and reduces as the extent of AD pathology progresses [60-63]. In our cohort, the extent of DNA damage (DNA strand breaks) was not altered in AD PBMCs relative to controls, and showed no trend with MMSE score. However, our measure of DNA damage is not specific to oxidative DNA damage. A more appropriate comparison from our study with the above studies would be the APE1 activity, since it is an indicator of DNA repair of oxidative lesions. The lower APE1 activity in the AD group of our study (Table 2) is consistent with high oxidative DNA damage; however, the slight inverse trend of APE1 activity with AD progression in our AD group is not consistent with reduced oxidative DNA damage as AD pathology progresses. However, there are many differences between our study and those studies, including our use of only mild to moderate AD, and our tissue source (PBMCs).

### Limitations of the study

A limitation in this study is that the population size was restricted due to practical issue associated with the large number of tests performed (five different assays, and a total of 14 parameters measured). Also, due to the multifaceted aspect of this study, the number of participants varied depending on which test was performed (see Table 2). However, our population size for each test is in accord with many peripheral blood cell biomarker studies which typically have a population number for each of the control and AD participants of less than 50; moreover, such studies typically involve only one parameter being measured [64, 65]. In addition, the bioenergetics and ROS analyses required live cells, which for practical reasons limits the population size. It is also possible that some tests have low sensitivity due to multiple biochemical sources that determine their levels (thus, the sum effect may be blunted). For example, our measure of DNA strand breaks indicates total DNA breaks that could arise from a number of endogenous or exogenous sources. It may be necessary to measure more specific damage, to detect differences in AD tissue, such as extent of oxidative lesions. Indeed, studies have reported elevated levels of oxidized bases in AD, by making use of lesion-specific endonucleases in the comet assay [2, 41]. Also, with regards to the gender comparisons of Figure 2A, the cohort is not ideal since it includes control and AD. We attempted to discern gender effects within the control and within the AD by stratifying for both gender and AD/control in Figure 2B, however the statistical power becomes reduced with the lower group population numbers; in fact, only APE1 activity retained statistical significance for gender effect (on both control and AD) and for significant alteration in AD (only retained in men).

We included patients with mild to moderate AD. We did not find any statistically significant associations between MMSE (global cognitive performance) and our molecular parameters. MMSE is a screening tool and, particularly in countries with a high level of education, many patients will score well during the early phases of AD, where it may not be sensitive to change. In further studies the performance of our molecular parameters in reflecting progression should be addressed in a longitudinal design, using other cognitive measures as well as global staging scales, such as the Clinical Dementia Rating Scale (CDR).

### Strengths of the study

The strengths of our study were that the diagnosis of AD was rigorous, our adjustments for age and gender were shown to be informative in that it suggests potential otherwise hidden alterations in AD PMBCs, and the techniques are potentially high throughput (especially determination of dNTP levels, since many frozen samples can be assayed simultaneously) which could make them useful as biomarkers for probable AD. Although our technique for measuring APE1 activity involved radioactivity (not ideal for high throughput biomarker testing) similar non-radioactive and equally sensitive BER assays for APE1 and other BER enzymes are being developed that have reduced costs, require very small amounts of protein extract, and that are easily automated [66, 67]. Our data is consistent with numerous reports of mitochondrial dysfunction in many neurological diseases, including AD, Parkinson's disease, Huntington's disease, and amyotrophic lateral sclerosis [68]. However, due to the cross-sectional design of this study, we cannot imply causality.

## METHODS

### Patient selection

Patients with mild to moderate AD were recruited from the Memory Clinic at Rigshospitalet, University of Copenhagen. The NINDS-ARDRA criteria were used for the diagnosis of probable AD. The diagnosis was established by clinical interview, neurological examination, cognitive tests, CT or MRI of the brain, and in selected cases, supplemental investigations. Patients who were unable to give informed consent were excluded. Healthy controls were age-matched volunteers, recruited from advertising, who had no major neurological or psychiatric disease and no significant cognitive deficits. In total, we recruited 53 patients with Alzheimer's disease with a mean age of 69.2 years and 30 age-matched healthy controls with a mean age of 66 years (Table 1). The sample size was calculated using power analysis. This analysis was performed with the assumption that the percentage of random missing data would be 5% as well as the other assumptions on expected means, standard deviations, α value (0.05) and tails (two-tailed test). The estimated means and standard deviations for the power analysis were derived from our previous study, in which these same parameters were measured in PBMCs of healthy participants [12]. Disease severity (i.e. AD progression) was determined by the mini-mental state examination (MMSE) to assess cognitive function including orientation, attention, recall, language and visuospatial functions. The MMSE consists of 11 items in a questionnaire, with a maximum score of 30 points. A score of 23 or lower indicates cognitive impairment, but is dependent on age and level of education [30].

### Isolation and storage of PBMCs

The isolation and freezing of the PBMCs for storage was performed as described previously [10, 12]. Briefly, PBMCs were isolated using BD Vacutainer Cell Preparation Tubes (CPT) containing sodium citrate (BD bioscience), according to the manufacturers protocol. PBMC isolation was performed on 8 ml of blood sample per participant, within 4 hours after blood withdrawal. Cells were counted by a cell counter (CASY^®^ cell counter, Roch Innovatis AG) and aliquoted for the various tests. Fresh cells were used for bioenergetics (Seahorse XP analyzer) and ROS measurements (flow cytometry). Cells were frozen in liquid nitrogen for later testing of dNTPs, DNA strand breaks and APE1 activity. For the dNTP assay, two million PBMCs were centrifuged in freezer tubes and the cell pellet was resuspended in 60% methanol and directly frozen and stored in liquid nitrogen. For the DNA strand breaks assay, one million PBMCs were centrifuged and the cell pellet snap frozen and stored in liquid nitrogen. For the APE1 activity assay, one million PBMCs were resuspended in freezing medium (50% fetal bovine serum, 40% DMEM, 10% DMSO) in freezing tubes, and then frozen first in −80°C in a pre-cooled (4°C) freezing container overnight (−1°C/min cooling rate) and then moved for long term storage to liquid nitrogen.

### Bioenergetics

The mitochondrial bioenergetic parameters were measured using the Seahorse Bioscience extracellular flux analyzer. This system is based on a pharmacological profiling approach that makes use of four added pharmaceutical modulators of mitochondrial electron transport chain fluxes, as described previously [12] and in Figure Supplemental S1 with representative OCR and ECAR profiles. Briefly, cells were seeded at 300,000 cells per well onto XF24 V7 cell culture microplates (Seahorse Bioscience, Billerica, MA) after coating the plates with Cell-Tak adhesive (BD Bioscience). We measured mitochondrial oxygen consumption rates (OCRs; indicators of mitochondrial respiration; specifically basal OCR, ATP turnover, reserve capacity, maximum capacity, and proton leak) and extracellular acidification rates (ECARs; indicators of glycolysis; specifically basal ECAR and glycolytic reserve), in the seeded PMBCs. This was accomplished by automated injection of pharmaceutical modulators of mitochondrial oxidative phosphorylation fluxes to the medium; the specific compounds added, sequentially, were 1 μM oligomycin, 0.3 μM FCCP and 2 μM antimycin A (Figure Supplemental S1). The chemical concentrations and PBMC seeding density were determined by titration. The reported level of each parameter from PBMCs of each participant was determined from the average of 10 wells.

### ROS production

The mitochondrial superoxide production was measured quantitatively by flow cytometry combined with MitoSOX red (Molecular Probes, Invitrogen). Two million PMBCs were pelleted and resuspended in 5 μM MitoSOX. After 10 minutes of incubation, cells were washed three times with PBS. All samples were prepared in triplicate. Determination of mitochondrial ROS was carried out using a FACScalibur (BD Bioscience). MitoSOX Red was excited at 488 nm and data collected at FSC, SSC and 585/42 nm (FL2) channels. The geometric mean fluorescence intensity values of the samples were obtained by subtracting the fluorescence of the control cells (not stained with MitoSOX) from the fluorescence of the MitoSOX stained cells.

### dNTPs

Whole cell levels of deoxyadenosine triphosphate (dATP), deoxycytidine triphosphate (dCTP), deoxyguanosine triphosphate (dGTP) and deoxythymidine triphosphate (dTTP) were determined using the DNA polymerase assay previously described [12]. Cellular dNTPs were extracted from two million PBMCs with 60% methanol. Radioactivity was measured in a Tri-Carb 2900TR liquid scintillation counter (Packard) and normalized to pmol/1×10^6^ cells using a standard curve of known dNTP concentrations. All samples were prepared in triplicate.

### Fluorometric detection of alkaline DNA unwinding

The levels of endogenous DNA strand breaks were measured using the Fluorometric detection of Alkaline DNA Unwinding (FADU) assay, as previously described [31, 32]. This assay measures both single and double strand DNA breaks but cannot distinguish between them. Decreased fluorescence signal as percentage of total fluorescence (= 100%) was used as the level of endogenous DNA damage; thus a higher value is indicative of a higher amount of DNA damage, in the form of DNA strand breaks. All samples were prepared in triplicate, at one million cells per well.

### APE1 incision assay

Cell extracts of one million cells were made and assayed as described previously [6]. The oligonucleotide DNA substrate used in this assay contains a tetrahydrofuran (THF) residue (= AP site), and has been used extensively by our group and others; the oligonucleotides are as follows: AP site strand, 5′-GAA CGA CTG T(AP site)A CTT GAC TGC TAC TGA T; complementary strand, 3′-CTT GCT GAC A CT GAA CTG ACG ATG ACT A. Reaction products were analyzed by electrophoresis under denaturing conditions; 20% acrylamide, 7 M urea, 1x TBE. Gel images were visualized by phosphorimaging (Typhoon 9410, Amersham Bioscience) and analyzed by ImageQuant 5.2 (Molecular Dynamics). Incision activity was determined, from the average of triplicate lanes (on separate gels), as the intensity of product bands relative to the combined intensities of substrate and product bands.

### Statistical analysis

To test for differences in the average values of each parameter between AD and control groups (Table 2) we used ANOVA, using GLM Univariate Analysis from IBM SPSS20 statistics software, New York, USA. In model 1, there were no adjustments for gender or age. In model 2 we adjusted for gender (men/women; categorical variable). In model 3 we adjusted for age (years; continuous variable), and in model 4 we adjusted for both gender and age. Gender effects on the parameters (Figure 2) were determined by two-tailed t-tests in GraphPad Prism 6 (La Jolla, CA, USA). For the association analysis of the parameter values with age (Figure 3) and with MMSE score (Figure 4), and association analysis among the biochemical parameters (Table S4), we used Pearson's correlation analysis with GraphPad Prism 6 software. *P* < .05 was considered significant. Several studies suggest that health behaviors such as physical activity, smoking and alcohol consumption are determinants of AD development [33]; in the case of smoking, the epidemiological studies suggest inconsistent results, suggesting either detrimental or even beneficial effects in AD [34]. However, health behaviors may be in the potential causal pathways between the biochemical parameters and AD development [35-37], and so they were not adjusted for. We did not adjust for hypertension since several participants (all controls) did not have blood pressure recorded.

### Ethical and safety aspects

This study was conducted according to the ethical principles of the Helsinki II declaration. The project was approved by the Ethical Review Committee of the Capital Region of Copenhagen (H-3-2011-137).

## Conclusion

We observed several biochemical defects in AD PBMCs, in the form of reduced basal respiration rate and proton leak, higher dATP level and lower APE1 activity. This data suggests that peripheral blood cells in AD patients display measurable defects in mitochondrial respiration, dNTP pool balance, and BER activity. Specifically, after statistical adjustment for gender, age, or both gender and age, our data suggest the following three concepts. 1. Proton leak and dATP level may be relevant as biomarkers without correction for gender or age: there was a significant difference in the average level of these parameters between control and AD without adjustment and moreover the adjustments had no striking effect on the strength of the outcome as indicated by the R^2^ and *P* values (see Table 2), 2. The utility of APE1 activity as a biomarker may be best if correction for gender is included: although the difference in average APE1 activity between control and AD was significant without correction (R^2^ = .124. *P* = .035), the outcome was much improved after correction for gender (R^2^ = .389, *P* = .003), 3. The utility of basal OCR as a biomarker appears to have the requirement for age correction: there was no significant difference in average basal OCR level between control and AD unless correction for age was performed (R^2^ = .092, *P* = .037). To validate the clinical utility of these results, an independent study is required of an equal or greater size to re-examine and further characterize the effects of AD or cognitive decline on mitochondrial bioenergetics, dNTP pools and DNA repair activity in AD.

## SUPPLEMENTAL INFORMATION FIGURE AND TABLES



## Abbreviations

AD: Alzheimer's disease
APE1: AP-endonuclease 1
BER: base excision repair
dTNPs: deoxyribonucleoside triphosphates
ECAR: extracellular acidification rate
MMSE: mini-mental state examination
OCR: oxygen consumption rate
OGG1: 8-oxoG DNA glycosylase 1
PBMC: peripheral blood mononuclear cells
ROS: reactive oxygen species
