# Supplementary material for: Defective mitochondrial respiration, altered dNTP pools and reduced AP endonuclease 1 activity in peripheral blood mononuclear cells of Alzheimer's disease patients
Source: Aging (Albany NY). 2015 Oct 21;7(10):793–810. doi: 10.18632/aging.100810 (PMC4637207; doi:10.18632/aging.100810)
Supplement: Supplementary file 1 [file aging-07-793-s001.pdf]

SUPPLEMENTAL INFORMATION

Representative OCR and ECAR profiles

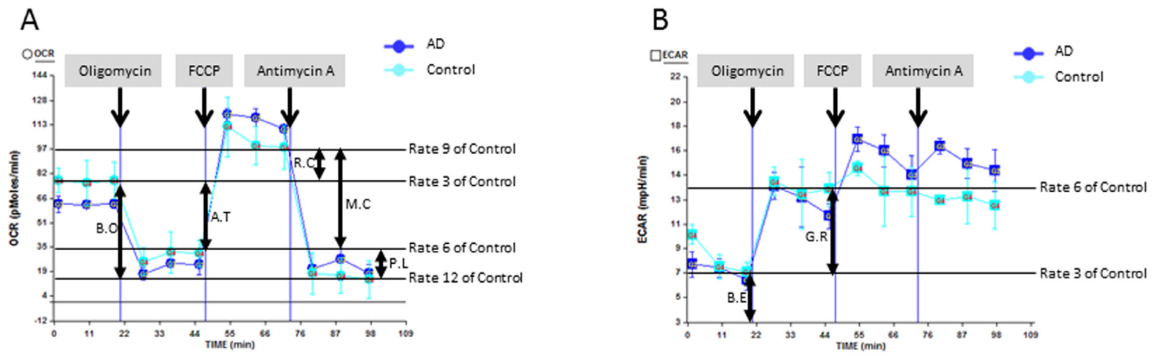

Figure S1. Representative outputs of the oxygen consumption rate (OCR) parameters (A) and extracellular acidification rate (ECAR) parameters (B) used in this report. The flux analyzer provides a non-invasive profile of the bioenergetic activity of cells in minutes, offering a physiological cell-based assay for determination of mitochondrial and glycolysis fluxes in a single experiment. For this illustration, data curves generated from randomly selected participants (one AD and one control that were measured simultaneously) in this study were used. Parameters signified by black double sided arrows, and the rates used to determine them, are indicated for the Control output. Rates were measured before and after sequential addition of oligomycin (1 µM), FCCP (0.3 µM) and antimycin A (2 µM), as indicated. ECAR was measured simultaneously with OCR. The third rate after the beginning of the run, and after the addition of each drug, was used for the OCR and ECAR measurements, to allow for equilibration (rates 3, 6, 9, and 12 as shown). Oligomycin inhibits ATP synthesis by blocking the proton channel of ATP synthase (Complex V). This results in a decrease in OCR to the extent to which the cells are using mitochondria to generate ATP. The remaining OCR is due to proton leak across the mitochondrial membrane and non-mitochondrial sources. There is a concomitant increase in ECAR as the cells revert to glycolysis to meet their energy demands. FCCP is an uncoupling agent that disrupts ATP synthesis by transporting hydrogen ions across the mitochondrial membrane instead of the proton channel of ATP synthase. This collapse of the mitochondrial membrane potential leads to a rapid consumption of oxygen without the generation of ATP. OCR increases due to this uncoupling. ECAR may increase beyond the existing ECAR as the cells continue to attempt to maintain their energy balance by using glycolysis to generate ATP. The final drug added was antimycin A, a complex III inhibitor. This causes the flow of electrons through the electron transport chain to cease. Therefore consumption of oxygen is drastically reduced. Again the ECAR may also increase slightly if necessary to maintain cellular energy balance. Abbreviations: B.O = Basal OCR; A.T = ATP turnover; R.C = Reserve capacity; M.C = Maximum capacity; P.L = Proton leak; B.E = Basal ECAR; G.R = Glycolytic reserve.

Supplemental Table S1. N, mean/SEM and 95% confidence interval for model 2 (adjusted for gender)

Variable		N	Mean (± SEM)	95% CI	R^2	*P*
Basal OCR	C	25	45.68 (3.24)	39.20-52.15	1	.071
	AD	43	38.18 (2.47)	33.25-43.12		
ATP turnover	C	25	37.05 (2.68)	31.7-42.40	0.06	.303
	AD	43	33.55 (2.04)	29.48-37.63		
Reserve capacity	C	25	22.03 (4.41)	13.23-30.83	0.053	.163
	AD	43	29.85 (3.36)	23.14-36.55		
Maximum capacity	C	25	67.71 (5.87)	55.99-79.42	0.061	.965
	AD	43	68.03 (4.47)	59.10-76.96		
Proton leak	C	25	8.61 (1.04)	6.53-10.70	0.086	.033*
	AD	43	5.75 (0.80)	4.16-7.34		
Basal ECAR	C	25	5.72 (0.50)	4.72-6.72	0.039	.287
	AD	43	6.40 (0.38)	5.64-7.16		
Glycolytic reserve	C	25	5.09 (0.50)	4.10-6.08	0.004	.735
	AD	43	5.30 (0.38)	4.54-6.06		
ROS production	C	16	8.67 (0.95)	6.75-10.59	0.176	.087
	AD	23	6.50 (0.79)	4.90-8.10		
dTTP	C	27	O.92 (0.17)	0.58-1.26	0.032	.204
	AD	28	1.23 (0.17)	0.89-1.56		
dATP	C	27	8.70 (0.65)	7.39-10.01	0.094	.035*
	AD	28	10.69 (0.64)	9.40-11.98		
dGTP	C	27	4.87 (0.42)	4.03-5.71	0.004	.666
	AD	28	5.13 (0.41)	4.30-5.95		
dCTP	C	27	1.51 (0.16)	1.18-1.84	0.044	.721
	AD	28	1.43 (0.16)	1.11-1.75		
DNA strand breaks	C	26	37.49 (1.71)	34.08-40.91	0.012	.749
	AD	44	38.19 (1.31)	35.57-40.81		
APE1 activity	C	18	53.47 (5.64)	42.00-64.93	0.389	.003*
	AD	18	27.93 (5.64)	16.47-39.40		

Abbreviations: OCR, oxygen consumption rate; ECAR, extracellular acidification rate; C, normal control participants; AD, Alzheimer's disease participants; N, population number; SEM, standard error of the mean.
Units: OCRs (Basal OCR, ATP turnover, Reserve capacity, Maximum capacity, Proton leak), pmol oxygen/min; ECARs (Basal ECAR, Glycolytic reserve), mpH/min; ROS production, fluorescence; dNTPs, pmol/million cells; DNA strand breaks, percent fluorescence; APE1 activity, percent incision.
*Significant difference ($P < .05$).

Supplemental Table S2. N, mean/SEM and 95% confidence interval for model 3 (adjusted for age)

Variable		N	Mean (± SEM)	95% CI	R^2	*P*
Basal OCR	C	25	46.63 (3.32)	40.00-53.25	0.092	0.037*
	AD	43	37.63 (2.51)	32.62-42.64		
ATP turnover	C	25	37.80 (2.73)	32.34-43.24	0.059	.183
	AD	43	33.12 (2.06)	29.00-37.25		
Reserve capacity	C	25	22.40 (4.52)	13.36-31.43	0.038	.213
	AD	43	29.63 (3.42)	22.80-36.46		
Maximum capacity	C	25	69.02 (6.05)	56.93-81.11	0.036	.820
	AD	43	67.26 (4.58)	58.12-76.40		
Proton leak	C	25	8.71 (1.07)	6.57-10.85	0.07	0.03*
	AD	43	5.70 (0.81)	4.07-7.31		
Basal ECAR	C	25	5.88 (0.51)	4.87-6.89	0.05	.508
	AD	43	6.31 (0.38)	5.54-7.07		
Glycolytic reserve	C	25	5.16 (0.51)	4.15-6.16	0.01	.873
	AD	43	5.26 (0.38)	4.50-6.02		
ROS production	C	16	8.87 (1.01)	6.81-10.92	0.089	.069
	AD	23	6.36 (0.84)	4.66-8.07		
dTTP	C	27	0.94 (0.17)	0.60-1.28	0.05	.264
	AD	28	1.21 (0.17)	0.87-1.54		
dATP	C	27	8.82 (0.65)	7.51-10.12	0.109	.060
	AD	28	10.58 (0.64)	9.29-11.86		
dGTP	C	27	4.91 (0.42)	4.08-5.75	0.024	.772
	AD	28	5.08 (0.41)	4.27-5.90		
dCTP	C	27	1.50 (0.17)	1.16-1.84	0.003	.830
	AD	28	1.44 (0.17)	1.11-1.77		
DNA strand breaks	C	26	37.64 (1.72)	34.20-41.08	0.001	.832
	AD	44	38.10 (1.32)	35.46-40.74		
APE1 activity	C	18	50.01 (6.66)	36.46-63.56	0.15	.059
	AD	18	31.39 (6.66)	17.84-44.94		

Abbreviations: OCR, oxygen consumption rate; ECAR, extracellular acidification rate; C, normal control participants; AD, Alzheimer's disease participants; N, population number; SEM, standard error of the mean.
Units: OCRs (Basal OCR, ATP turnover, Reserve capacity, Maximum capacity, Proton leak), pmol oxygen/min; ECARs (Basal ECAR, Glycolytic reserve), mpH/min; ROS production, fluorescence; dNTPs, pmol/million cells; DNA strand breaks, percent fluorescence; APE1 activity, percent incision.
*Significant difference ($P < .05$)

Supplemental Table S3. N, mean/SEM and 95% confidence interval for model 4 (adjusted for gender and age)

Variable		N	Mean (± SEM)	95% CI	R^2	*P*
Basal OCR	C	25	47.00 (3.23)	40.55-53.44	0.157	.023*
	AD	43	37.41 (2.44)	32.54-42.28		
ATP turnover	C	25	38.06(2.68)	32.71-43.41	0.111	.140
	AD	43	32.97 (2.02)	28.92-37.01		
Reserve capacity	C	25	22.68 (4.51)	13.67-31.69	0.061	.242
	AD	43	29.46 (3.41)	22.65-36.27		
Maximum capacity	C	25	69.68 (5.90)	57.90-81.46	0.102	.710
	AD	43	66.88 (4.46)	57.97-75.78		
Proton leak	C	25	8.78 (1.07)	6.65-10.91	0.095	.024*
	AD	43	5.65 (0.81)	4.04-7.26		
Basal ECAR	C	25	5.87 (0.50)	4.86-6.88	0.071	.492
	AD	43	6.31 (0.38)	5.55-7.07		
Glycolytic reserve	C	25	5.16 (0.51)	4.14-6.17	0.012	.880
	AD	43	5.26 (0.38)	4.49-6.02		
ROS production	C	16	8.76 (0.98)	6.78-10.74	0.181	.080
	AD	23	6.44 (0.81)	4.80-8.08		
dTTP	C	27	0.94 (0.17)	0.59-1.28	0.05	.272
	AD	28	1.21 (0.17)	0.87-1.55		
dATP	C	27	8.79 (0.66)	7.47-10.10	0.116	.055
	AD	28	10.61 (0.65)	9.31-11.91		
dGTP	C	27	4.92 (0.42)	4.08-5.80	0.025	.797
	AD	28	5.08 (0.41)	4.25-5.90		
dCTP	C	27	1.51 (0.17)	1.18-1.85	0.044	.711
	AD	28	1.43 (0.16)	1.10-1.75		
DNA strand breaks	C	26	37.54 (1.73)	34.09-41.00	0.013	.780
	AD	44	38.16 (1.33)	35.51-40.81		
APE1 activity	C	18	52.83 (5.74)	41.14-64.52	0.399	.006*
	AD	18	28.57(5.74)	16.88-40.26		

Abbreviations: OCR, oxygen consumption rate; ECAR, extracellular acidification rate; C, normal control participants; AD, Alzheimer's disease participants; N, population number; SEM, standard error of the mean.
Units: OCRs (Basal OCR, ATP turnover, Reserve capacity, Maximum capacity, Proton leak), pmol oxygen/min; ECARs (Basal ECAR, Glycolytic reserve), mpH/min; ROS production, fluorescence; dNTPs, pmol/million cells; DNA strand breaks, percent fluorescence; APE1 activity, percent incision.
*Significant difference ($P < .05$).

Supplemental Table S4. Associations among the biochemical parameters

Variable		ATP turn.	Res. Cap.	Max cap.	Proton leak	Basal ECAR	Glyc. res.	ROS prod.	dTTP	dATP	dGTP	dCTP	DNA Strand breaks	APE1 act.
	P	< .0001*	.2437	< .0001*	< .0001*	.0539	< .0001*	.1223	.2463	.7332	.6316	.7586	.2439	.9776
**Basal OCR**	r	0.9378	0.1433	0.6721	0.6136	0.2349	0.4686	0.2483	-0.159	0.04701	0.06611	-0.0424	-0.1432	-0.00485
	N	68	68	68	68	68	68	40	55	55	55	55	68	36
	P		.0805	< .0001*	.007*	.0087*	< .0001*	.0545	.4157	.8842	.3888	.7475	.2710	.7955
**ATP turn.**	r		0.2135	0.6895	0.3243	0.316	0.5887	0.3064	-0.112	-0.02009	0.1185	-0.04441	-0.1354	0.04474
	N		68	68	68	68	68	40	55	55	55	55	68	36
	P			< .0001*	.8189	< .0001*	< .0001*	.7586	.1085	.7086	.3671	.6148	.0769	.6688
**Res. cap.**	r			0.8291	-0.02828	0.5648	0.5778	0.05015	0.2188	0.05155	0.124	-0.06936	-0.216	-0.0738
	N			68	68	68	68	40	55	55	55	55	68	36
	P				.0068*	< .0001*	< .0001*	.2511	.6061	.6426	.3531	.5891	.0463*	.7321
**Max cap.**	r				0.3254	0.5552	0.697	0.1858	0.07108	0.06399	0.1276	-0.07444	-0.2425	-0.0591
	N				68	68	68	40	55	55	55	55	68	36
	P					.9575	.9499	.8789	.5859	.0639	.5641	.8871	.8433	.6037
**Proton leak**	r					0.006582	0.00776	-0.02488	-0.07508	0.2516	0.07946	0.0196	-0.02442	-0.08949
	N					68	68	40	55	55	55	55	68	36
	P						< .0001*	.3052	.0788	.1681	.1923	.0630	.1178	.7579
**Basal ECAR**	r						0.6952	0.1663	0.2391	0.1885	0.1785	0.2524	-0.1914	-0.05322
	N						68	40	55	55	55	55	68	36
	P							.2948	.6823	.9708	.6104	.5299	.0711	.1020
**Glyc. res.**	r							0.1698	0.05645	-0.00505	0.07024	0.08652	-0.2203	-0.2769
	N							40	55	55	55	55	68	36
	P								.2008	.1744	.7026	.2410	.7174	.4033
**ROS prod.**	r								-0.2066	-0.2191	-0.06228	-0.1897	-0.05906	0.1436
	N								40	40	40	40	40	36
	P									.2021	.0004*	< .0001*	.8978	.1610
**dTTP**	r									0.1747	0.4618	0.6101	0.01772	0.2387
	N									55	55	55	55	36
	P										.2781	.9449	.8040	.1152
**dATP**	r										0.1488	-0.00953	0.03425	-0.2671
	N										55	55	55	36
	P											.0024*	.7926	.1841
**dGTP**	r											0.4009	0.03628	0.2265
	N											55	55	36
	P												.2839	.4251
**dCTP**	r												-0.1471	0.1372
	N												55	36
	P													.2167
**DNA Strand breaks**	r													0.211
	N													36

Abbreviations: OCR, oxygen consumption rate; ECAR, extracellular acidification rate; ATP turn. = ATP turnover; Res. Cap. = Reserve capacity; Max cap. = Maximum capacity; Glyc. Res. = Glycolytic reserve; ROS prod = ROS production; APE1 act. = APE1 activity. Units: OCRs (Basal OCR, ATP turnover, Reserve capacity, Maximum capacity, Proton leak), pmol oxygen/min; ECARs (Basal ECAR, Glycolytic reserve), mpH/min; ROS production, fluorescence; dNTPs, pmol/million cells; DNA strand breaks, percent fluorescence; APE1 activity, percent incision.

*Significant difference ($P < .05$). P values are written to four decimal places in this table since several associations were < .0001.
